# Prevalence, species diversity, and antimicrobial resistance profiles of *Campylobacter* spp. among children under five years of age with acute diarrhea in Ouagadougou, Burkina Faso

**DOI:** 10.1128/spectrum.01243-26

**Published:** 2026-07-06

**Authors:** Alimatou Héma, Issa Tondé, Jean Zongo, Marguerite Edith Malatala Nikiema, Evariste Bako, Ange Badjo, Mamadou Tamboura, Absatou Ky-Ba, Moussa Sangaré, Abdoul-Salam Ouédraogo, Mahamoudou Sanou

**Affiliations:** 1 Université Joseph KI-ZERBO (UJKZ), Ouagadougou, Burkina Faso; 2 Laboratory of Bacteriology and Virology, Centre Hospitalier Universitaire de Tengandogo (CHU-T), Ouagadougou, Burkina Faso; 3 Laboratory of Bacteriology and Virology, Centre Hospitalier Universitaire Pédiatrique Charles de Gaulle (CHUP-CDG), Ouagadougou, Burkina Faso; 4 Laboratory of Virology and Plant Biotechnology, Institut de l’Environnement et de Recherches Agricoles (INERA), Ouagadougou, Burkina Faso; 5 Department of Biochemistry and Microbiology, Centre Universitaire de Tenkodogo, Tenkodogo, Burkina Faso; 6 Emerging and Re-emerging Pathogens Laboratory (LAPATHER), Université Nazi Boni (UNB), Bobo-Dioulasso, Burkina Faso; 7 Centre Hospitalier Universitaire de Bogodogo (CHU-B), Ouagadougou, Burkina Faso; 8 International Center of Excellence in Research in Mali (ICER-Mali), Faculty of Medicine and Odonto-Stomatology (FMOS), Techniques and Technologies of Bamako (USTTB), University of Sciences, Bamako, Mali; 9 Laboratory of Bacteriology and Virology, Centre Hospitalier Universitaire Sourô Sanou (CHUSS), Bobo-Dioulasso, Burkina Faso; 10 Centre Muraz, Bobo-Dioulasso, Burkina Faso; 11 Burkina Faso National Reference Laboratory for Antimicrobial Resistance (LNR-RAM), Centre Hospitalier Universitaire Sourô Sanou (CHUSS), Bobo-Dioulasso, Burkina Faso; 12 International Institute of Science and Technologies (IISTech), Ouagadougou, Burkina Faso; South China Sea Institute of Oceanology Chinese Academy of Sciences, Guangdong, China

**Keywords:** *Campylobacter*, diarrhea, children, antimicrobial resistance, multidrug resistance, One Health, Burkina Faso

## Abstract

*Campylobacter* species are major causes of pediatric gastroenteritis worldwide, yet their burden remains underestimated in West Africa due to diagnostic challenges. This study aimed to determine the prevalence, species diversity, and antimicrobial resistance (AMR) profiles of *Campylobacter* spp. among children under 5 years of age with acute diarrhea in Ouagadougou, Burkina Faso. From 1 May 2023 to 30 April 2024, 383 stool samples were collected at the Centre Hospitalier Universitaire Pédiatrique Charles de Gaulle. Samples were analyzed using standard culture on Karmali selective agar and biochemical identification via the API Campy system. AMR testing was performed using the disk diffusion method. *Campylobacter* spp. were detected in 33 samples (8.6% prevalence). Infection was significantly associated with the 12–23 months age group (48.5%; *P* < 0.05). *Campylobacter jejuni* was the most common species (42.4%), followed by *C. coli* (21.2%) and *C. fetus* subsp. *fetus* (21.2%). Other species included *C. hyointestinalis* (6.1%), *C. sputorum* (6.1%), and *C. lari* (3.0%). Alarmingly, 100% of isolates were resistant to ampicillin, erythromycin, and tetracycline. High resistance rates were also observed for amoxicillin-clavulanic acid (93.9%) and ciprofloxacin (75.8%). Gentamicin showed the highest susceptibility (57.6%). All isolates (100%) were classified as multidrug-resistant (MDR). These results highlight a high prevalence of MDR *Campylobacter* in Ouagadougou, with total resistance to erythromycin, the first-line treatment. The identification of *C. fetus* at 42°C suggests a high environmental and zoonotic pressure. These findings underscore the urgent need for enhanced genomic surveillance and updated clinical management guidelines for pediatric diarrhea in the region.

**IMPORTANCE:**

This study highlights the burden and antimicrobial resistance of *Campylobacter* spp. among children under five with acute diarrhea in Burkina Faso. Culture-based detection identified *Campylobacter* in 8.6% of children, confirming its role as an important but under-recognized cause of pediatric diarrheal disease. High levels of antimicrobial resistance, including complete resistance to erythromycin, raise concerns about the effectiveness of commonly used treatment options. The identification of multiple *Campylobacter* species, including *C. fetus*, suggests a broader diversity of circulating strains and possible zoonotic transmission pathways. These findings provide valuable baseline data to strengthen antimicrobial resistance surveillance, improve diagnostic practices, and support evidence-based treatment strategies in West African settings.

## INTRODUCTION

Diarrheal diseases remain a major global public health problem, particularly among children under 5 years of age, accounting for approximately 1.7 billion episodes and 443,832 deaths annually (1). The burden is disproportionately concentrated in Sub-Saharan Africa (2), where repeated enteric infections contribute not only to acute mortality but also to long-term morbidity, including growth faltering, impaired cognitive development, and environmental enteric dysfunction (3, 4).

Among bacterial enteropathogens, *Campylobacter* spp. are recognized as leading causes of zoonotic gastroenteritis worldwide (5). *Campylobacter jejuni* and *Campylobacter coli* are the most clinically relevant species associated with childhood diarrhea (6). Beyond acute gastroenteritis, *Campylobacter* infections have been associated with important post-infectious sequelae, most notably Guillain-Barré Syndrome (GBS), an acute immune-mediated polyradiculoneuropathy that can lead to flaccid paralysis and significant morbidity (7, 8). Molecular mimicry between *Campylobacter* lipooligosaccharides and peripheral nerve gangliosides is thought to play a central role in GBS pathogenesis (9). In addition to acute infection, emerging evidence links *Campylobacter*-associated gut dysbiosis with chronic inflammatory responses and disruption of intestinal microbiota homeostasis (10–12).

Poultry production represents a major reservoir for zoonotic transmission (13, 14). Contaminated poultry meat and poor hygiene practices facilitate environmental exposure, particularly in urban and peri-urban settings (15, 16). Furthermore, rising antimicrobial resistance (AMR) among *Campylobacter* isolates is an increasing clinical challenge, particularly resistance to macrolides and fluoroquinolones (17, 18), which are first-line therapies for severe cases (9, 19).

In low- and middle-income countries (LMICs), the epidemiology of *Campylobacter* infection remains poorly characterized due to limited surveillance infrastructure and reliance on conventional culture-based diagnostics (20, 21). Molecular studies have demonstrated substantially higher detection rates compared with culture-based approaches, suggesting that historical prevalence estimates may substantially underestimate the true burden of enteric infections (20, 21). Although culture-independent diagnostic methods have markedly improved the detection of enteropathogens in both clinical and surveillance settings, important limitations must be considered. In particular, while molecular assays enhance the detection of *Campylobacter* spp., they do not distinguish between viable and non-viable organisms, and therefore a positive PCR result does not necessarily indicate active infection. In addition, genus-level detection may include *Campylobacter* spp. that are not recoverable using standard culture conditions optimized for *Campylobacter jejuni* (*C. jejuni*) and *Campylobacter coli* (*C. coli*), or that may be less pathogenic or non-pathogenic (22). These factors may contribute to discrepancies between molecular and culture-based estimates and complicate both clinical interpretation and epidemiological assessments (22).

Despite the superior sensitivity of molecular diagnostics, including genomic approaches, culture-based methods remain indispensable for phenotypic AMR surveillance (21, 23). Stool samples are inherently polymicrobial, and the isolation of individual microorganisms is therefore required to accurately link specific pathogens to their corresponding resistance phenotypes. The recovery of viable isolates is a fundamental prerequisite for standardized antimicrobial susceptibility testing, which is essential for tracking emerging resistance patterns, informing local antibiograms, and guiding effective empirical treatment (23).

In Burkina Faso, previous hospital-based studies using culture methods reported low *Campylobacter* prevalence rates ranging from 2% to 3% (24, 25). For instance, Sangaré et al. (24) detected *Campylobacter* in 2.3% of 1,246 patients with enteritis in Ouagadougou (24), while Bonkoungou et al. (25) identified the pathogen in 2% of 283 children under five in a multi-pathogen study of diarrhea (25). In contrast, a recent molecular epidemiological study conducted in Bobo-Dioulasso and Dano reported substantially higher prevalence rates across all age groups (20), suggesting that the burden of *Campylobacter* infection may be considerably underestimated when relying solely on culture-based diagnostics.

Despite this, phenotypic antimicrobial susceptibility data from pediatric clinical isolates in Burkina Faso remain scarce, limiting the ability to inform evidence-based treatment strategies and monitor emerging resistance patterns.

This study aimed to determine the prevalence, species distribution, and antimicrobial resistance profiles of *Campylobacter* spp. among children under 5 years presenting with diarrhea in Ouagadougou.

## MATERIALS AND METHODS

### Study design and setting

This was a prospective, hospital-based cross-sectional study conducted between 1 May 2023 and 30 April 2024. The study was based at the “Centre Hospitalo-Universitaire Pédiatrique Charles de Gaulle (CHUP-CDG)” in Ouagadougou, Burkina Faso. CHUP-CDG is a tertiary-level referral hospital specializing in pediatric medicine and serves a diverse urban and peri-urban population. Laboratory analyses were performed at the hospital’s Bacteriology-Virology Department.

### Study population and eligibility

The study population included children aged 0–59 months who presented to the pediatric emergency or outpatient departments with acute or persistent diarrhea. Diarrhea was defined as the passage of three or more loose or liquid stools per 24-h period (1). To minimize the risk of false-negative culture results, children who had received antimicrobial therapy within the 72 h prior to admission were excluded.

### Sample size determination

The sample size was calculated using the single population proportion formula:


$$n=\frac{Z^{2}\times P(1-p)}{d^{2}}$$

where *Z* is the standard normal deviate at the 95% confidence level (1.96), *p* is the expected prevalence, and *d* is the margin of error (0.05). Given the variability in previous reports (20, 24, 25), a conservative 50% prevalence assumption was used to maximize statistical precision, resulting in a target of 384. A total of 383 children were ultimately enrolled and provided stool samples for culture. This represents 99.7% of the calculated target and was considered negligible with respect to statistical precision.

### Stool culture and bacterial identification

Fresh stool specimens were collected in sterile containers and immediately inoculated onto Karmali selective agar (Oxoid CM0935B) supplemented with *Campylobacter* Growth Supplement (Oxoid SR0232E). Plates were incubated at 42°C for 48 h under microaerophilic conditions (5% O_2_, 10% CO_2_, and 85% N_2_) using the bioMérieux GENBOX Microaer system. It should be noted that incubation at 42°C selectively favors thermophilic *Campylobacter* spp. (*C. jejuni* and *C. coli*); not all *C. fetus* isolates grow at 42°C, as this subspecies grows optimally between 25°C and 37°C (26), and its recovery under these conditions may therefore be incidental.

Presumptive *Campylobacter* colonies identified by their small, grayish, translucent, or spreading moist morphology were confirmed microscopically as gram-negative, motile, “seagull-wing” curved rods.

Biochemical confirmation included positive oxidase and catalase reactions. Final species-level identification was performed using the API Campy system (bioMérieux), a 20-test miniaturized biochemical strip with database (https://apiweb.biomerieux.com). *Escherichia coli* (ATCC 25922) and *Staphylococcus aureus* (ATCC 25923) served as quality control strains for media selectivity and biochemical accuracy.

### Antimicrobial susceptibility testing (AST)

Antimicrobial susceptibility testing was performed on all confirmed *Campylobacter* isolates using the disk diffusion method on Mueller-Hinton agar supplemented with 5% blood (MH-F), in accordance with the recommendations of the CA-SFM. The antibiotics tested included ampicillin (10 µg), amoxicillin–clavulanic acid (20/10 µg), erythromycin (15 µg), ciprofloxacin (5 µg), tetracycline (30 µg), and gentamicin (10 µg).

Plates were incubated under microaerophilic conditions at 35 ± 2°C for 24 h, with extended incubation up to 44 h when necessary to ensure adequate growth.

Multidrug resistance (MDR) was defined as non-susceptibility to at least one agent in three or more antimicrobial classes.

Interpretation of inhibition zone diameters for *Campylobacter jejuni* and *Campylobacter coli* was performed according to the CA-SFM/EUCAST clinical breakpoint tables (version 14.0, 2024). For non-jejuni/coli species (*C. fetus*, *C. hyointestinalis*, *C. sputorum*, and *C. lari*), where standardized clinical interpretive breakpoints are not explicitly defined in EUCAST or CA-SFM guidelines, available epidemiological cutoff values (ECOFFs) were utilized to distinguish wild-type from non-wild-type strains. In the absence of validated clinical breakpoints, *C. jejuni*/*coli* criteria were applied as a pragmatic surrogate for erythromycin, tetracycline, ampicillin, and amoxicillin-clavulanic acid, consistent with published evidence supporting their applicability across major human *Campylobacter* species (27). For ciprofloxacin, where clinical breakpoints are lacking, interpretations relied strictly on these ECOFFs (28); accordingly, ciprofloxacin results for these minor species were reported as non-wild type when applicable, rather than clinically resistant, and were interpreted with caution.

### Data management and statistical analysis

Data were captured using KoboCollect v6.2.1 and analyzed using R software. Categorical variables are presented as frequencies and percentages. Differences in isolation rates between age groups and sexes were compared using the chi-square test or Fisher’s exact test, as appropriate. A *P* value < 0.05 was considered statistically significant.

## RESULTS

### Demographic characteristics

**Fig 1:**
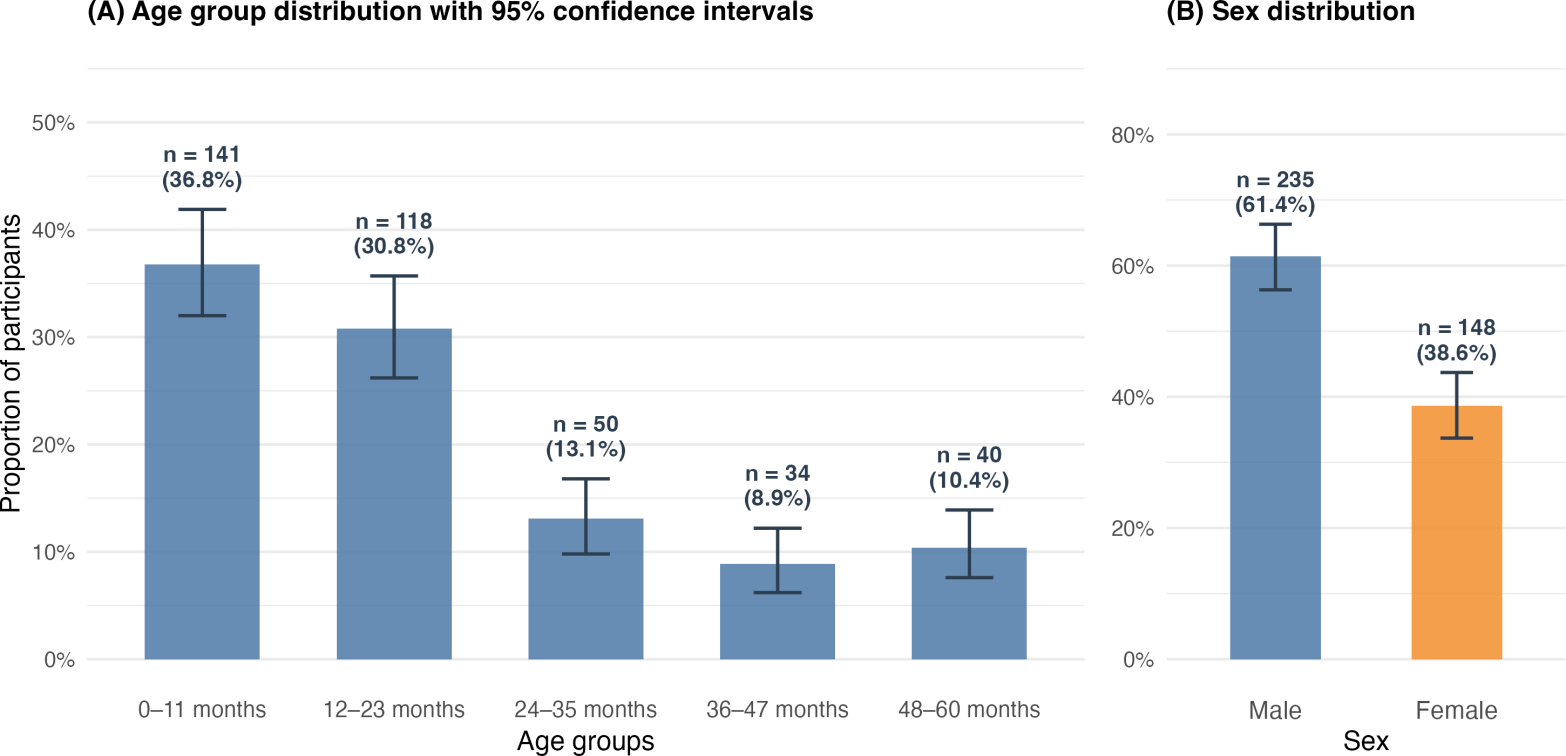
Demographic characteristics of children under 5 years of age presenting with acute diarrhea. (**A**) Age group distribution. (**B**) Sex distribution. Error bars represent 95% confidence intervals calculated using the exact binomial (Clopper-Pearson) method.

The study included 383 children under 5 years of age clinically diagnosed with acute diarrhea. Of the enrolled participants, 235 (61.4%) were male. The median age of participants was 15 months (IQR: 9–24), with most children (67.6%) under 24 months of age (0–23 months). Demographic details are further detailed in Fig. 1.

### Study population and *Campylobacter* prevalence

Of the 383 children enrolled and meeting eligibility criteria, all provided a stool specimen suitable for culture. *Campylobacter* spp. were isolated from 33 samples, corresponding to a culture-based prevalence of 8.6% (95% CI: 6.0–11.9%). Among positive cases, 54.5% (*n* = 18) were male (*P* = 0.600).

**Fig 2:**
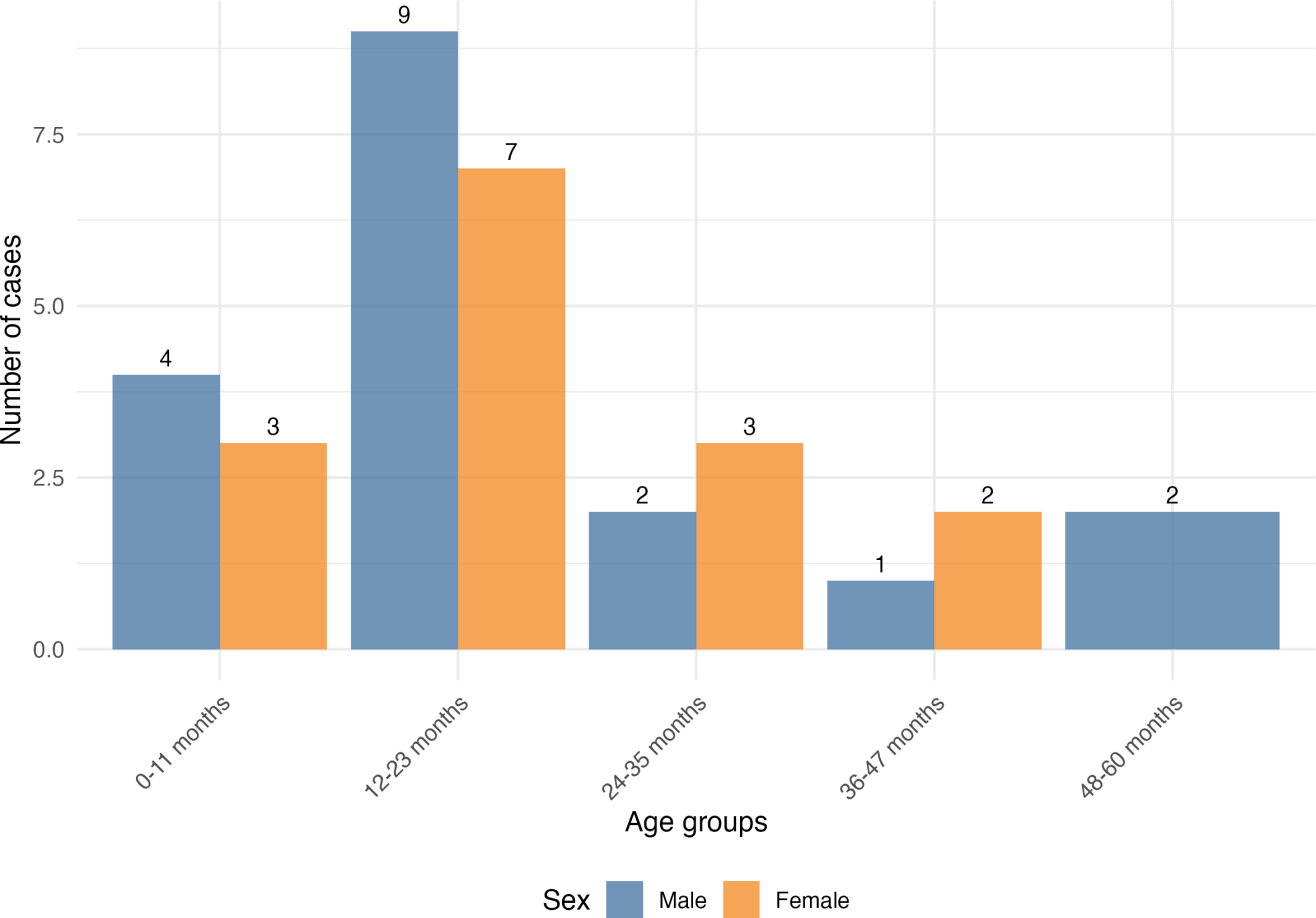
Distribution of *Campylobacter* cases by age group and sex.

Age-stratified analysis showed a significantly higher isolation rate among children aged 12–23 months compared with all other age groups (*P* = 0.043). This age group represented nearly half of all positive cases (48.5%, *n* = 16) (Fig. 2).

### Species distribution

Of the 33 confirmed isolates, thermophilic *Campylobacter* species (*C. jejuni* and *C. coli*) collectively accounted for the majority (63.6%, *n* = 21). *C. jejuni* was the most frequently identified species, followed by *C. coli*.

**Fig 3:**
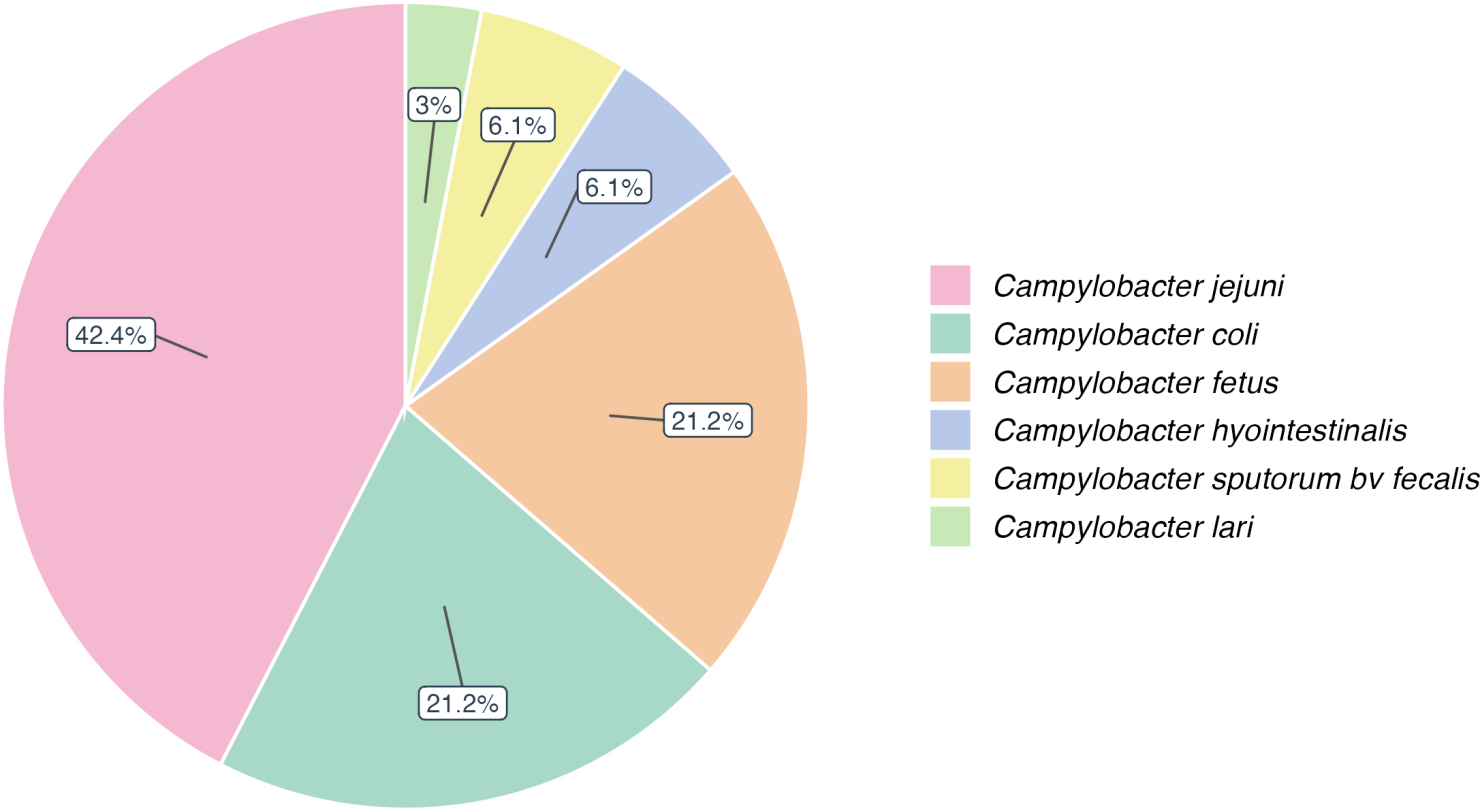
Distribution of *Campylobacter* isolates.

Non-thermophilic species represented (36.4%, *n* = 12) of isolates, with *Campylobacter fetus* subsp. *fetus* being the most common within this group. The detailed species distribution is presented in Fig. 3.

### Antimicrobial susceptibility profiles

Antimicrobial susceptibility testing of the 33 isolates demonstrated 100% (33/33; 95% CI: 89.4–100%) resistance to ampicillin, erythromycin, and tetracycline. High levels of phenotypic resistance were also observed for amoxicillin-clavulanic acid 93.9% (31/33; 95% CI: 79.8–99.3%).

**Fig 4:**
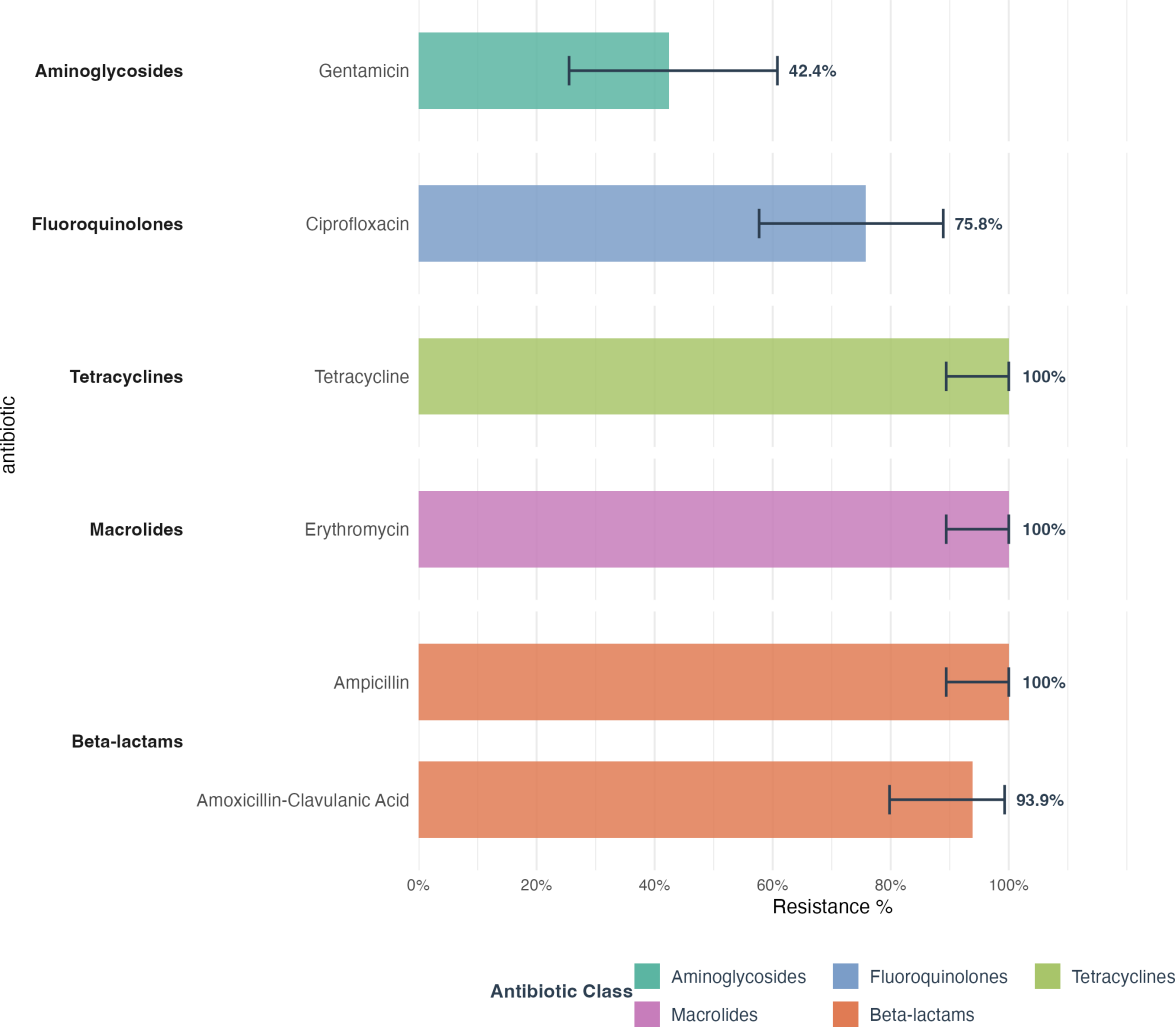
Antimicrobial resistance (AMR) rates in *Campylobacter* isolates.

For the agents with intermediate interpretive categories, ciprofloxacin resistance was 75.8% (25/33; 95% CI: 57.7–88.9%), with 12.1% (4/33) of isolates testing intermediate and 12.1% (4/33) testing susceptible. Gentamicin showed the highest level of residual activity, with 57.6% (19/33) of isolates remaining susceptible, while 42.4% (14/33; 95% CI: 25.5–60.8%) were resistant (Table 1). A visual summary of these resistance frequencies, grouped by antimicrobial class, is provided in Fig. 4.

**TABLE 1:** Antimicrobial resistance (AMR) profiles of *Campylobacter* isolates (*N* = 33)

Antimicrobial agent	Resistant *n* (%)	95% CI^*a*^	Intermediate *n* (%)	Susceptible *n* (%)
Ampicillin	33 (100%)	89.4–100%	0 (0%)	0 (0%)
Amoxicillin-clavulanic acid	31 (93.9%)	79.8–99.3%	0 (0%)	2 (6.1%)
Ciprofloxacin	25 (75.8%)	57.7–88.9%	4 (12.1%)	4 (12.1%)
Erythromycin	33 (100%)	89.4–100%	0 (0%)	0 (0%)
Gentamicin	14 (42.4%)	25.5–60.8%	0 (0%)	19 (57.6%)
Tetracycline	33 (100%)	89.4–100%	0 (0%)	0 (0%)

^*a*^ 95% Confidence intervals (CIs) calculated using the exact binomial method based on total resistant isolates out of *N* = 33*.*

### Multidrug resistance profiles

**Fig 5:**
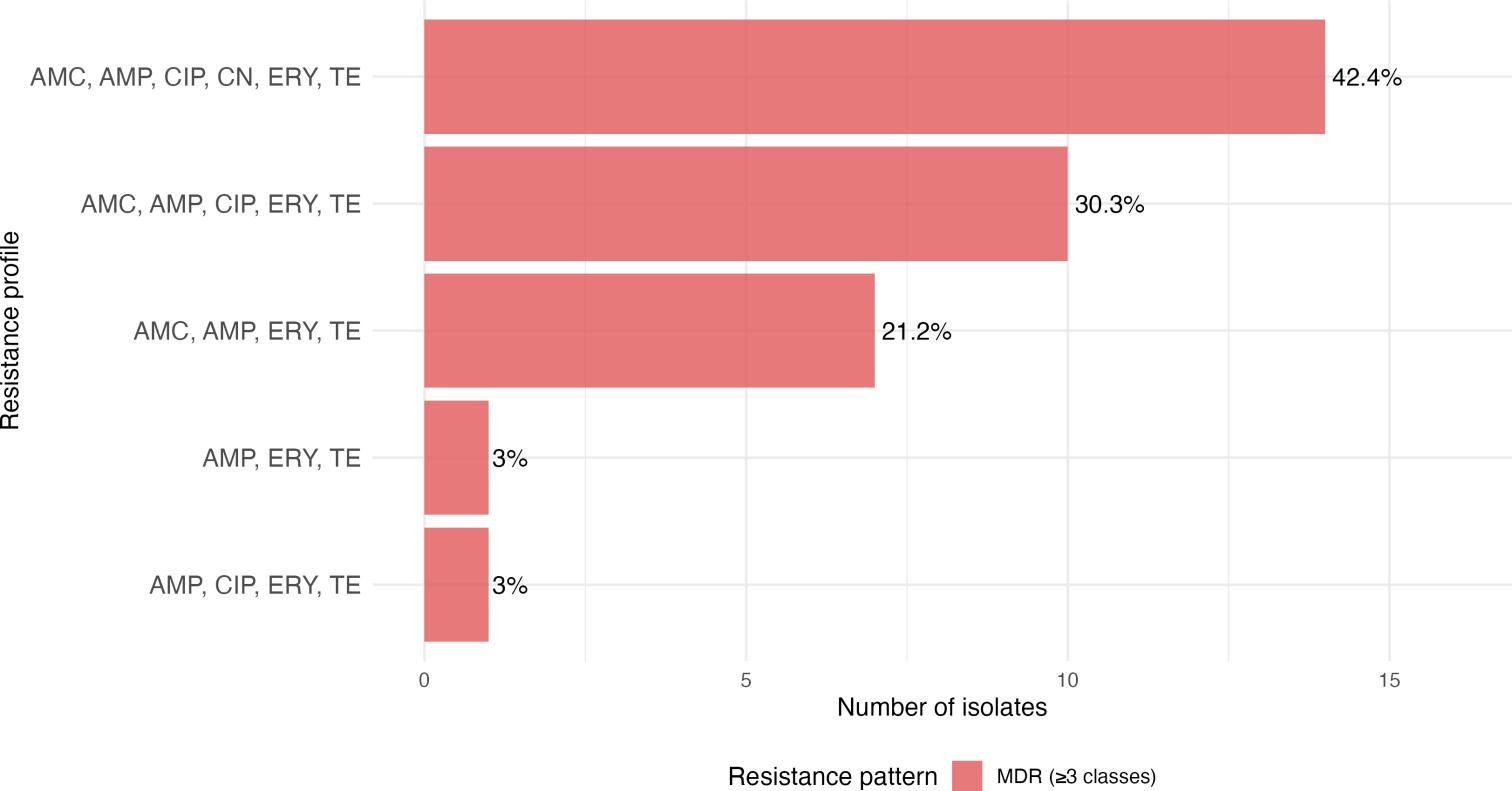
Multidrug resistance profiles of *Campylobacter* isolates (*n* = 33). Bars represent the number and percentage of isolates exhibiting specific resistance patterns. AMC, amoxicillin-clavulanic acid; AMP, ampicillin; CIP, ciprofloxacin; CN, gentamicin; ERY, erythromycin; TE, tetracycline.

Multidrug resistance (MDR), defined as resistance to at least one agent in three or more antimicrobial classes, was observed in all isolates (100%, 33/33). The most frequent resistance profile involved simultaneous non-susceptibility to ampicillin, amoxicillin-clavulanic acid, ciprofloxacin, gentamicin, erythromycin, and tetracycline (Fig. 5).

### Species-specific resistance patterns

Resistance to ampicillin, erythromycin, and tetracycline was uniformly 100% (33/33) across all isolated species (Table 2). High rates of resistance to amoxicillin-clavulanic acid were also observed. Among *C. coli* isolates, 28.6% (2/7) were susceptible, while the remaining 71.4% (5/7) were resistant. All isolates from other species were resistant (100%, 33/33).

**TABLE 2:** Species-specific antimicrobial resistance (AMR) patterns^*b*^

Antimicrobial agent	*C. jejuni* (*n* = 14)	*C. coli* (*n* = 7)	*C. fetus^a^* (*n* = 7)	*C. hyointestinalis* (*n* = 2)	*C. sputorum^a^* (*n* = 2)	*C. lari* (*n* = 1)	Total
Ampicillin	14 (100.0%) (76.8–100)	7 (100.0%) (59.0–100)	7 (100.0%) (59.0–100)	2 (100.0%) (15.8–100)	2 (100.0%) (15.8–100)	1 (100.0%) (2.5–100)	33 (100.0%) (89.4–100)
Amoxicillin-clavulanic acid	14 (100.0%) (76.8–100)	5 (71.4%) (29.0–96.3)	7 (100.0%) (59.0–100)	2 (100.0%) (15.8–100)	2 (100.0%) (15.8–100)	1 (100.0%) (2.5–100)	31 (93.9%) (79.8–99.3)
Ciprofloxacin	10 (71.4%) (41.9–91.6)	6 (85.7%) (42.1–99.6)	7 (100.0%) (59.0–100)	0 (0.0%) (0.0–84.2)	2 (100.0%) (15.8–100)	0 (0.0%) (0.0–97.5)	25 (75.8%) (57.7–88.9)
Erythromycin	14 (100.0%) (76.8–100)	7 (100.0%) (59.0–100)	7 (100.0%) (59.0–100)	2 (100.0%) (15.8–100)	2 (100.0%) (15.8–100)	1 (100.0%) (2.5–100)	33 (100.0%) (89.4–100)
Gentamicin	3 (21.4%) (4.7–50.8)	4 (57.1%) (18.4–90.1)	6 (85.7%) (42.1–99.6)	0 (0.0%) (0.0–84.2)	1 (50.0%) (1.3–98.7)	0 (0.0%) (0.0–97.5)	14 (42.4%) (25.5–60.8)
Tetracycline	14 (100.0%) (76.8–100)	7 (100.0%) (59.0–100)	7 (100.0%) (59.0–100)	2 (100.0%) (15.8–100)	2 (100.0%) (15.8–100)	1 (100.0%) (2.5–100)	33 (100.0%) (89.4–100)

^*a*^ *C. fetus = Campylobacter fetus *subsp.* fetus*; *C. sputorum = Campylobacter sputorum* bv. fecalis.

^*b*^ Values are presented as *n* (%) (95% confidence interval) calculated via the Clopper-Pearson exact binomial method.

Ciprofloxacin resistance was widespread but variable: it was 100% in *C. fetus* subsp. *fetus* (7/7) and *C. sputorum bv. fecalis* (2/2), and high in *C. coli* (85.7%, 6/7) and *C. jejuni* (71.4%, 10/14). All isolates of *C. hyointestinalis* (2) and *C. lari* (1) were susceptible.

Gentamicin demonstrated the highest overall *in vitro* activity. Resistance was absent in *C. hyointestinalis* (0/2) and *C. lari* (0/1), low in *C. jejuni* (21.4%, 3/14) and *C. sputorum bv. fecalis* (50.0%, 1/2), and more pronounced in *C. coli* (57.1%, 4/7) and *C. fetus* subsp. *fetus* (85.7%, 6/7).

## DISCUSSION

A culture-based prevalence of 8.6% of *Campylobacter* spp. among children under five presenting with acute diarrhea highlights the continued contribution of this pathogen to pediatric enteric infections in Burkina Faso. This estimate is consistent with reports from similar low-resource settings and confirms that *Campylobacter* remains a significant, yet often under-recognized, enteropathogen in pediatric populations.

The predominance of thermophilic species, particularly *Campylobacter jejuni* and *Campylobacter coli*, aligns with global epidemiological patterns, although the substantial proportion of non-thermophilic species underscores a broader diversity of circulating strains in this setting. A particular concern is the high level of antimicrobial resistance observed, including universal multidrug resistance and complete resistance to commonly used antibiotics such as ampicillin, erythromycin, and tetracycline. These findings raise serious implications for clinical management and underscore the urgent need for strengthened antimicrobial stewardship and continuous surveillance of *Campylobacter* infections in similar contexts.

The 8.6% *Campylobacter* prevalence observed in this pediatric cohort is three- to fourfold higher than rates reported in earlier culture-based studies from Ouagadougou (2.0–2.3%) (24, 25). This difference may reflect improvements in sample handling and the use of validated selective media under controlled microaerophilic conditions. However, our estimate remains substantially lower than PCR-based detection rates (25%) reported in recent molecular studies conducted in both urban (Bobo-Dioulasso) and rural (Dano) settings in Burkina Faso (20), highlighting the limited sensitivity of culture for this fastidious pathogen, which requires viable organisms for growth (23).

Furthermore, the ability of *Campylobacter* to enter a viable but non-culturable (VBNC) state under stress may further contribute to under-detection by culture (29).

Consequently, our findings likely underestimate the true burden of *Campylobacter* infection.

The age distribution, with a peak prevalence among children aged 12–23 months (48.5%), aligns with the weaning period, a critical window during which maternally derived immunity declines and exposure to contaminated food, water, and animal reservoirs increases (30).

While *Campylobacter jejuni* (42.4%) and *Campylobacter coli* (21.2%) predominated, the proportion of *Campylobacter fetus* subsp. *fetus* (21.2%) is notable. Globally, the primary reservoirs of *Campylobacter fetus* subsp. *fetus* are the gastrointestinal tracts of cattle and sheep, with exposure occurring principally through foods of animal origin and cross-contaminated foodstuffs (26). Although *Campylobacter fetus* is classically regarded as an opportunistic pathogen affecting immunocompromised or elderly individuals (26), its detection in stool samples from young children in this cohort, which corroborates previous reports of *Campylobacter fetus* in local poultry in Burkina Faso (31), suggests that zoonotic exposure in the urban and peri-urban areas of Ouagadougou may extend to the entire pediatric population. This finding is particularly noteworthy given that *Campylobacter fetus* is rarely recovered in routine surveillance, partly because standard thermophilic culture conditions are not optimized for its isolation (26), meaning its clinical burden in this population is likely underestimated.

The antimicrobial resistance (AMR) profiles observed in this study raise substantial clinical concerns. Universal resistance to ampicillin, erythromycin, and tetracycline effectively precludes their use for empirical therapy. Macrolide resistance in *Campylobacter* is most commonly mediated by point mutations (A2074G and A2075G) in the 23S rRNA gene, particularly A2075G, or by acquisition of the *erm(B*) gene, both of which confer stable, high-level resistance (17, 19). In contrast to other enteric pathogens such as *Salmonella*, where susceptibility may vary according to macrolide structure, a single 23S rRNA mutation in *Campylobacter* confers high-level resistance across macrolides, resulting in markedly elevated MICs irrespective of ring size (32).

The complete loss of erythromycin susceptibility observed in this study is of major concern, given its role as the first-line treatment for severe campylobacteriosis (33–35), and highlights a critical therapeutic gap in this setting. This resistance may reflect selective pressure from macrolide use, including azithromycin, which is widely administered in mass drug distribution programs and may contribute to cross-resistance (33, 36).

The observed multidrug resistance (MDR) rate of 100% further suggests the involvement of broad resistance mechanisms, such as the *CmeABC* efflux pump (37). Although gentamicin retained partial activity (57.6% susceptibility), its parenteral administration and potential toxicity limit its use in routine pediatric care. Overall, these findings emphasize the urgent need to strengthen antimicrobial stewardship and to implement integrated surveillance strategies in both clinical and agricultural settings.

A key strength of this study is the generation of culture-confirmed, phenotypic antimicrobial susceptibility data using standardized CA-SFM/EUCAST methodologies in a region where such data remain critically scarce. While molecular diagnostics offer higher sensitivity for pathogen detection, the recovery of viable isolates is an indispensable prerequisite for phenotypic resistance characterization, the only approach that directly informs local antibiogram development and empirical treatment guidelines. The prospective design, and the use of validated selective media under controlled microaerophilic conditions further strengthen the reliability of the microbiological data generated. Beyond the primary focus on thermophilic species, this study also provides rare descriptive data on non-thermophilic *Campylobacter* species including *C. fetus*, *C. hyointestinalis*, *C. sputorum*, and *C. lari* recovered from a pediatric clinical setting in West Africa. Such species are systematically underreported in surveillance studies from low- and middle-income countries, largely because standard culture protocols are not optimized for their recovery. The detection of *C. fetus* in particular, accounting for 21.2% of isolates and previously documented in local poultry reservoirs (31) provides preliminary but valuable evidence of zoonotic exposure to non-thermophilic species in urban Ouagadougou and highlights the potential clinical relevance of these organisms in pediatric diarrheal disease, a dimension rarely captured in routine surveillance. These findings, while exploratory, contribute to a more complete picture of the *Campylobacter* spp. diversity circulating in this setting underscores the importance of broad-spectrum species identification in clinical microbiology practice.

Several limitations should, however, be acknowledged. First, logistical and national supply chain challenges restricted our total enrollment to 383 children during the study period, which may impact the precision of our overall *Campylobacter* prevalence estimate. Consequently, this yielded a relatively small number of recovered viable isolates (*n* = 33), thereby reducing the statistical power and precision of our species-specific antimicrobial resistance profiles, as reflected in the wide 95% confidence intervals for less frequent species. Second, as a single-center, hospital-based study conducted at a tertiary referral facility, our findings likely reflect a clinically selected population with more severe or treatment-refractory diarrheal illness and may not be generalizable to community settings or primary healthcare levels.

Third, the use of Karmali agar incubated at 42°C, a condition selectively optimized for thermophilic *Campylobacter* species, may have resulted in the incidental and potentially non-representative recovery of non-thermophilic species, particularly *C. fetus* (26). For these isolates, isolation rates may underestimate true prevalence, and antimicrobial susceptibility results carry an additional interpretive caveat: no validated EUCAST/CA-SFM clinical breakpoints currently exist for *C. fetus* subsp. *fetus* or other non-thermophilic species. For ciprofloxacin specifically, only epidemiological cutoff values (ECOFFs) that identify the boundary of the wild-type population but do not predict therapeutic outcome have been proposed (28). Consequently, our reliance on ECOFFs for these minor strains introduces a degree of diagnostic uncertainty relative to absolute clinical outcomes; ciprofloxacin results for *C. fetus* subsp. *fetus* isolates (21.2% of the total, *n* = 7) reflect phenotypic non-wild-type status rather than clinically confirmed resistance, and species-specific resistance profiles should be interpreted with this nuance in mind.

Fourth, the absence of molecular validation tools, such as PCR amplification or sequencing for specific gyrA and 23S rRNA point mutations, precludes us from directly identifying the genetic determinants driving these phenotypes. Furthermore, the lack of whole-genome sequencing limits the ability to characterize mobile genetic elements or assess clonal relatedness with strains circulating in animal reservoirs (13). Finally, AST quality control relied on *Escherichia coli* ATCC 25922 alone; the absence of a *Campylobacter*-specific reference strain (e.g., *C. jejuni* ATCC 33560) is acknowledged as a limitation, as species-specific QC strains provide more direct validation of zone diameter interpretations for fastidious microaerophilic organisms, a constraint inherent to the resource-limited context of this study.

Nevertheless, these phenotypic data offer immediate, actionable insight for empirical pediatric prescribing patterns and diagnostic stewardship in a region where baseline surveillance data remain critically sparse.

### Conclusion

Pediatric campylobacteriosis in Ouagadougou is characterized by a substantial disease burden and alarming levels of multidrug resistance. The observed complete resistance to erythromycin, the current first-line therapy, underscores the urgent need to reassess empirical treatment guidelines in this setting. These findings highlight the importance of integrated One Health surveillance to monitor antimicrobial resistance across the human, animal, and environmental interface, alongside strengthened antimicrobial stewardship to reduce selective pressure in both clinical and agricultural sectors.

From a microbiological standpoint, this study also illustrates the diagnostic gap inherent in relying exclusively on 42°C incubation protocols. The incidental detection of non-thermophilic species, notably *C. fetus,* which accounted for 21.2% of isolates, suggests that the true diversity of clinically relevant *Campylobacter* species in this setting may be substantially underestimated by standard thermophilic culture conditions.

Future surveillance studies in Burkina Faso and across West Africa should use parallel incubations at 37°C and 42°C to systematically isolate both thermophilic and non-thermophilic *Campylobacter* species. Notably, *C. fetus*, which grows optimally at 37°C but inconsistently at 42°C (26), might otherwise go undetected, which would limit our understanding of species diversity and its clinical and zoonotic implications.

## Data Availability

The data sets generated and analyzed during the current study are available from the corresponding author upon reasonable request.
